# First mammography screening participation and breast cancer incidence and mortality in the subsequent 25 years: population based cohort study

**DOI:** 10.1136/bmj-2025-085029

**Published:** 2025-09-24

**Authors:** Ziyan Ma, Wei He, Yuqi Zhang, Xinhe Mao, José Tapia, Per Hall, Keith Humphreys, Kamila Czene

**Affiliations:** 1 Department of Medical Epidemiology and Biostatistics, Karolinska Institutet, Stockholm, Sweden; 2 Department of Nutrition and Food Hygiene, Children’s Hospital, Zhejiang University School of Medicine, National Clinical Research Center for Child Health, Hangzhou, Zhejiang, China; 3 Chronic Disease Research Institute, School of Public Health, School of Medicine, Zhejiang University, Hangzhou, Zhejiang, China; 4 Department of Oncology, Södersjukhuset, Stockholm, Sweden

## Abstract

**Objective:**

To determine whether women who did not attend their first mammography screening invitation have a long term risk of poor screening adherence and breast cancer outcomes.

**Design:**

Population based cohort study.

**Setting:**

Stockholm, Sweden.

**Participants:**

432 775 women who received invitations to the Swedish Mammography Screening Programme between 1991 and 2020 and were initially invited at either 50 years of age or 40 years of age.

**Main outcome measures:**

Screening adherence, breast cancer incidence, tumour characteristics, and breast cancer mortality tracked through linkage to multiple Swedish national registers, with follow-up until 2023 (up to 25 year follow-up period). Cumulative breast cancer incidences were calculated from first screening participation. Cox proportional hazards models estimated hazard ratios for breast cancer mortality; logistic regression models assessed associations with tumour characteristics by odds ratios.

**Results:**

During a total of 4 940 375 person years of follow-up, 16 059 new cases of breast cancer were documented. Among women invited to their first mammography screening, 32.1% (n=138 760) did not participate. These non-participants were persistently less likely to attend subsequent screenings and were more likely have symptom detected, advanced stage breast cancer diagnosed. Specifically, compared with first screening participants, non-participants had an odds ratio of 1.53 (95% confidence interval 1.24 to 1.88) for stage III cancer (160 (4.1%) *v* 266 (2.9%) cases) and 3.61 (2.79 to 4.68) for stage IV cancer (150 (3.9%) *v* 105 (1.2%) cases). During a total of 6 818 686 person years of follow-up, 1603 deaths from breast cancer were documented. Non-participation at first screening was also associated with significantly higher breast cancer mortality, with a 25 year cumulative mortality of 9.9 per 1000 versus 7.0 per 1000 for participants (adjusted hazard ratio 1.40, 95% confidence interval 1.26 to 1.55). By contrast, the 25 year breast cancer incidence was similar between groups (7.8% in participants versus 7.6% in non-participants), suggesting that the elevated mortality among first screening non-participants likely reflects delayed detection rather than increased incidence.

**Conclusions:**

This study shows that first screening non-participants represent a large population at long term risk of dying from breast cancer, providing an opportunity for targeted interventions to improve adherence to screening and thereby decrease mortality risk.

## Introduction

Despite significant advances in screening for and treatment of breast cancer leading to reduced mortality rates over recent decades,^1^ late stage breast cancer remains a persistent challenge. Stage III and IV breast cancer accounts for 8-22% of all cases,^2^ significantly contributing to breast cancer related deaths.^3^ This burden of advanced disease highlights the critical need to identify populations at high risk early, enabling targeted interventions that could prevent delayed diagnoses and improve breast cancer outcomes.

Mammography screening continues to be the most effective tool for early detection of breast cancer, with many studies showing its role in reducing later stage cancer diagnoses and mortality.^4^ ^5^ ^6^ ^7^ ^8^ Although later stage diagnosis is more frequent among breast cancers diagnosed in women who did not attend the latest screening,^9^ a knowledge gap exists regarding the long term implications of earlier screening behaviours, particularly first screening participation.

First mammography screening could be a crucial time point for prevention of adverse breast cancer outcomes. If early screening behaviour is predictive of later stage diagnosis and mortality risk, it could provide a valuable opportunity to identify populations at high risk decades before adverse outcomes occur. This early identification would create a substantial time window for intervention, potentially altering the trajectory of breast cancer outcomes.

Using data from nearly half a million women with up to 25 years of follow-up through the Swedish Mammography Screening Register and linked Swedish national registers, we investigated the association between participation at the first invited mammography screening and future breast cancer outcomes. These outcomes included subsequent breast cancer screening behaviour, breast cancer incidence (by mode of detection), tumour characteristics, and breast cancer mortality.

## Methods

### Data sources

The study was designed as a cohort study, using data initially sourced from the Swedish Mammography Screening Register.^10^ This register has systematically collected information on all invitations to and participations in the population based mammography screening programme in Stockholm since 1989. Before July 2005, the programme exclusively invited women aged 50-69 years at 24 month intervals. From July 2005 to 2012, the programme expanded to include women aged 40-49 years with 18 month invitation intervals, while maintaining biennial invitations for those aged 50-69 years. From 2013, the programme further extended to include women aged 70-74 years, who were also invited at biennial intervals. Because invitation is determined solely by age and residency, the cohort includes all eligible women in Stockholm County who were invited to mammography screening, thereby representing the full target population rather than a sampled subset. The average participation rate stands at around 70%.^10^

By using the unique personal identity number, we achieved virtually complete linkage with other national registers.^11^ The proportion of missingness was <5% for most variables.^10^ ^12^ ^13^ ^14^ ^15^ ^16^ ^17^ ^18^ The study complies with the Strengthening the Reporting of Observational Studies in Epidemiology (STROBE) guidelines for cohort studies.^19^

### Study population

**Fig 1:**
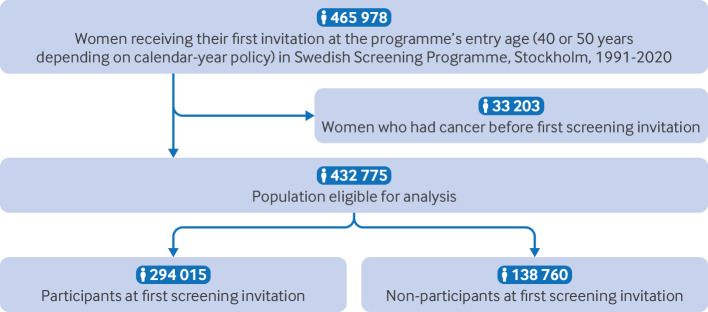
Flowchart of study enrolment

We initially identified women who had ever received an invitation to mammography screening between 1991 and 2020. To ensure methodological rigour and standardise baseline exposure to screening, we restricted our cohort to women who received their first screening invitation precisely at age 50 (before July 2005) or at age 40 (from July 2005 onward). This approach minimised confounding from heterogeneous screening histories, and we excluded women who received their first invitations at other ages to maintain cohort uniformity. By using the personal identity number, we linked the population with the Swedish Cancer Register, and we excluded women with cancer diagnosed before their first screening invitation. Figure 1 includes more detailed information on the selection process.

### First screening participation (exposure)

We defined the first screening as the initial screening invitation entry in the Swedish Mammography Screening Register at either 50 years of age or 40 years of age (after 2005 only). The register recorded participation statuses as either “participated” or “not participated.”^10^ Women who received an invitation were initially assigned an individual appointment time, which could be rescheduled by phone, letter, or email. Importantly, women who rescheduled and subsequently attended their screening were classified as “participated,” with the actual screening date recorded as the time of participation. The “not participated” category was reserved for women who either actively cancelled without rescheduling or did not attend their appointment without notification. This classification approach ensured that participation status reflected true engagement with screening rather than adherence to a specific appointment slot. Among women who attended, the time from first invitation to actual screening participation had a median of 25 (interquartile range 21-35) days; 5% participated on the same day as their invitation, and 95% participated within 72 days.

### Outcomes

This study focused on four outcomes: overall participation in screening, incidence of breast cancer (overall and by mode of detection), tumour characteristics of breast cancer, and breast cancer mortality. These are described below.

#### Cumulative screening participation

We defined cumulative screening participation in terms of participation in up to nine screenings following the first screening, with information sourced from the Swedish Mammography Screening Register.

#### Breast cancer incidence

Information on breast cancer incidence came from the Swedish Cancer Register.^20^ This legally mandated, virtually complete national registry documents all cancers diagnosed in Sweden since 1958, including information on diagnosis dates and cancer types classified according to the ICD-7 (international classification of diseases, seventh revision) (ICD-7 code for breast cancer: 170).^20^

We classified breast cancers into three mutually exclusive modes of detection on the basis of the patients’ most recent screening participation status: screen detected cancer, diagnosed during routine mammography screenings; interval cancer, diagnosed symptomatically after a negative screening result but before the next scheduled screening round; and symptom detected cancer after missed screening, diagnosed symptomatically in women who did not attend their most recently invited screening. This classification directly links the mode of detection to screening behaviour and allows us to examine whether first screening non-participants show persistent non-attendance patterns that consequently lead to a higher risk of symptom detected, potentially later stage disease.

We defined follow-up for breast cancer incidence as starting at the first round of screening invitation and ending at breast cancer diagnosis (outcome of interest), other cancer diagnosis, two years after those women passed screening age (that is, 71 or 76 years old), death, emigration, 25 years after the start of follow-up, or December 2020, whichever occurred first.

#### Breast cancer tumour characteristics

Information on tumour characteristics came from the Stockholm Regional Cancer Center Register and the National Quality Register for Breast Cancer, which includes details on invasiveness (in situ/invasive), tumour size (T; <20 mm, 20-50 mm, >50 mm), regional lymph node involvement (N; no/yes), and distant metastasis (M; no/yes). Because T, N, and M components were recorded according to the TNM edition in force at diagnosis (4th-8th editions), we reclassified every case with the 7th edition of the American Joint Committee on Cancer and International Union for Cancer Control stage group algorithm, assigning stage I, II, III, or IV.^21^ The anatomical thresholds defining these four stages have remained constant across editions, so this uniform rule set yields a stage variable that is fully comparable over the entire 1991-2020 study period.

#### Mortality

We extracted data on all cause mortality and cause specific mortality from the Swedish Cause of Death Register.^22^ This high quality, virtually complete register documents all deaths in Sweden since 1952, including information on time of death and cause specific codes.^22^ Breast cancer mortality was coded as 174 (ICD-9) and C50 (ICD-10), whereas non-breast cancer mortality refers to deaths from other causes. We defined the start of follow-up for cause specific mortality as the first round of screening invitation and end of follow-up as death from breast cancer, death from other causes, emigration, 25 years after the start of follow-up, or December 2023, whichever occurred first.

### Covariates

We collected data on population characteristics from various population registers at the time of the first screening invitation. Educational attainment, income, and marital status came from the Longitudinal Integrated Database for Health Insurance and Labour Market Studies. Information on people born in Sweden came from the Total Population Register. The Multi-generation Register provided data on the number of children and age at first childbirth, and the National Patient Register, as well as the Charlson Comorbidity Index, supplied information on alcohol related and obesity related diseases.^23^ We gathered information on family history of cancer from cancer diagnosis records of first degree relatives in the Swedish Cancer Register and the Multi-generation Register. We selected these variables on the basis of availability and because they represent factors known or suspected to be associated with non-participation in screening from epidemiological literature,^24^ and they thus constitute potential confounders.

### Statistical analysis

We presented baseline characteristics by first screening participation status, with numbers and percentages. For understanding the connections of participation behaviours between screening rounds, we calculated the cumulative participation across 10 screenings, stratified by first screening participation. We applied logistic regression models to assess the associations between first screening participation and subsequent participation in the second and 10th screenings.

We used the Aalen-Johansen estimator to calculate the 25 year cumulative incidence of screen detected breast cancer, interval breast cancer, and symptom detected breast cancer after missed screening, accounting for each other as competing risks. For women in whom breast cancer was diagnosed, we used logistic or multinomial logistic regression models to assess the relation between first screening non-participation (independent variable) and tumour characteristics (dependent variables).

We used Cox proportional hazards models to analyse associations between first screening participation and breast cancer specific mortality; we also examined corresponding associations with non-breast cancer mortality and all cause mortality as comparisons. We used the Schoenfeld residual test to check the proportionality assumption of the Cox proportional hazards model and found no violation of the assumption. We reported hazard ratios and 95% confidence intervals.

To assess the robustness of our findings, we did sensitivity analyses for both the tumour characteristics and mortality outcomes. For tumour characteristics, we modelled tumour size and TNM stage as ordinal outcomes by using cumulative logistic regression. For breast cancer mortality analysis, we repeated the main Cox regression models with the following key modifications: using attained age as the underlying time scale^25^ (replacing time since first screening invitation) to correct for differences in intervals between women aged 40-49 (invited every 18 months) and ≥50 years (invited every 24 months); excluding women with strong family histories of breast cancer (n=2885) to reduce potential confounding from opportunistic mammography screening outside the organised programme, which in Sweden typically involves women referred for genetic counselling; excluding cases of breast cancer diagnosed within three months after the latest screening invitation was missed (n=151) to evaluate whether our results might be influenced by women who declined the invitation because they were already undergoing diagnostic investigation for symptoms (for example, mammography or magnetic resonance imaging) and subsequently received a breast cancer diagnosis; and excluding screen detected cancers recalled owing to clinical findings (n=418) to evaluate potential misclassification of symptomatic cancers captured through screening.

The above statistical analyses all included two models: a partially adjusted model adjusted for age and calendar year and a fully adjusted model that additionally included the other covariates mentioned above, unless otherwise stated. In all multivariable regression models, we treated missing values for the covariates as a separate group. All statistical tests were two sided tests, and we considered results to be statistically significant at P<0.05. We used R version 4.2.2 for all analyses.

### Patient and public involvement

Patients and the public were not directly involved in the design, conduct, or reporting of this study. However, the research was informed by longstanding engagement with women participating in screening programmes. It was highly motivated by the priorities and experiences of women involved in breast cancer screening, with the overarching aim of enhancing screening practices to help to reduce breast cancer mortality.

## Results

### Baseline characteristics

The cohort consisted of 432 775 women, of whom 294 015 (68.9%) attended their first screening and 138 760 (32.1%) did not attend (fig 1). Table 1 shows the population characteristics of the study cohort at the first invited mammography screening.

**Table 1:** Characteristics of population stratified by participation at first invited mammography screening. Values are numbers (percentages)

Characteristics	Total population (n=432 775)	First mammography screen		
		Participants (n=294 015)	Non-participants (n=138 760)	
Age at first invitation:				
40 years	205 990 (47.6)	141 940 (48.3)	64 050 (46.2)	
50 years	226 785 (52.4)	152 075 (51.7)	74 710 (53.8)	
Year of first invitation:				
1991-2000	120 511 (27.8)	81 213 (27.6)	39 298 (28.3)	
2001-10	159 508 (36.9)	108 021 (36.7)	51 487 (37.1)	
2011-20	152 756 (35.3)	104 781 (35.6)	47 975 (34.6)	
Educational attainment:				
	57 951 (13.6)	35 402 (12.1)	22 549 (16.8)	
10-12 years	163 040 (38.2)	111 352 (38.1)	51 688 (38.4)	
	205 656 (48.2)	145 290 (49.7)	60 366 (44.8)
Missing	6128 (1.4)	1971 (0.7)	4157 (3.0)
Income level:			
Quarter 1	67 333 (15.8)	36 575 (12.5)	30 758 (22.9)
Quarter 2	75 022 (17.6)	49 396 (16.9)	25 626 (19.0)
Quarter 3	121 600 (28.5)	85 023 (29.1)	36 577 (27.2)
Quarter 4	162 692 (38.1)	121 050 (41.4)	41 642 (30.9)
Missing	6128 (1.4)	1971 (0.7)	4157 (3.0)
Born in Sweden:			
Yes	307 521 (71.1)	218 519 (74.3)	89 002 (64.1)
No	125 254 (28.9)	75 496 (25.7)	49 758 (35.9)
Marital status:			
Single	111 596 (25.8)	72 798 (24.8)	38 798 (28.1)
Married	235 554 (54.5)	168 323 (57.3)	67 231 (48.6)
Divorced	84 731 (19.6)	52 482 (17.9)	32 249 (23.3)
Missing	894 (0.2)	412 (0.1)	482 (0.3)
Parity:			
None	74 790 (17.3)	44 628 (15.2)	30 162 (21.7)
1-2	254 292 (58.8)	179 302 (61.0)	74 990 (54.0)
≥3	103 693 (24.0)	70 085 (23.8)	33 608 (24.2)
Age at first child*:			
≤30 years	256 404 (71.6)	177 711 (71.3)	78 693 (72.5)
>30 years	101 581 (28.4)	71 676 (28.7)	29 905 (27.5)
Alcohol related diseases:			
Yes	6016 (1.4)	2829 (1.0)	3187 (2.3)
No	426 759 (98.6)	291 186 (99.0)	135 573 (97.7)
Obesity related diseases:			
Yes	8882 (2.1)	5732 (1.9)	3150 (2.3)
No	423 893 (97.9)	288 283 (98.1)	135 610 (97.7)
Charlson Comorbidity Index:			
0	397 478 (91.8)	270 822 (92.1)	126 656 (91.3)
1-2	32 684 (7.6)	21 702 (7.4)	10 982 (7.9)
≥3	2613 (0.6)	1491 (0.5)	1122 (0.8)
Cancer family history:			
Parent	124 767 (28.8)	88 521 (30.1)	36 246 (26.1)
Sibling	17 372 (4.0)	12 004 (4.1)	5368 (3.9)
Parent and sibling	15 720 (3.6)	10 926 (3.7)	4794 (3.5)
None	274 916 (63.5)	182 564 (62.1)	92 352 (66.6)
Breast cancer family history:			
Mother	21 143 (4.9)	14 850 (5.1)	6 293 (4.5)
Sibling	4113 (1.0)	2730 (0.9)	1383 (1.0)
Mother and sibling	456 (0.1)	268 (0.1)	188 (0.1)
None	407 063 (94.1)	276 167 (93.9)	130 896 (94.3)

Information was collected at time of first screening invitation.

* Among women with children (n=357 985).

### Overall screening participation

**Fig 2:**
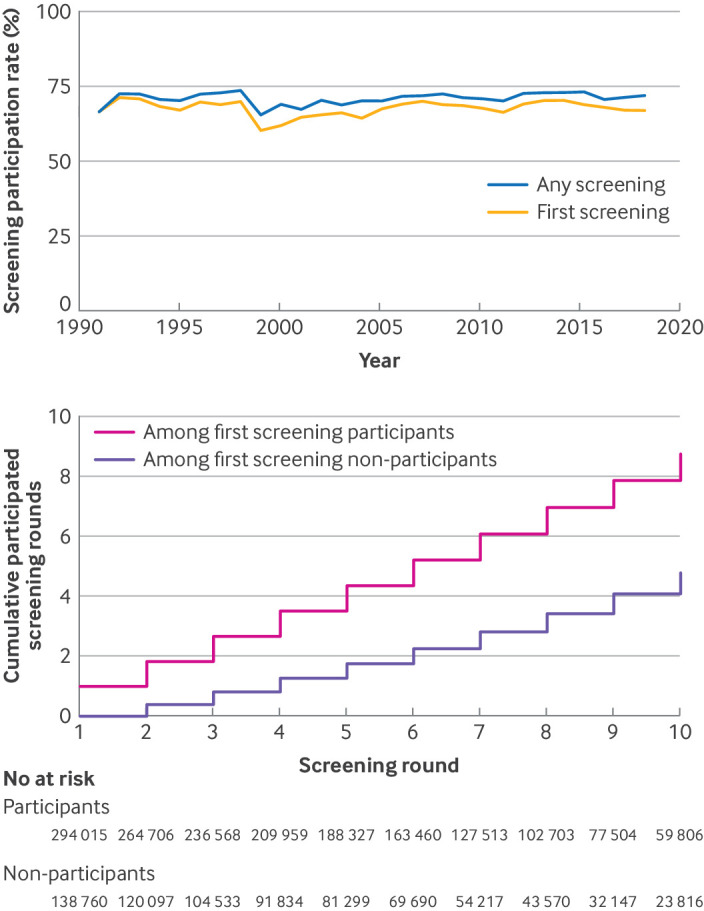
Overview of screening participation over calendar period (top) and screening rounds following participation at first invited mammography screening (bottom). Top: screening participation rate (%) by calendar year, for any screening (number of participants divided by number of invited in that calendar year) and for first screening (number of first screening participants divided by number of first screening invitation in that calendar year). Bottom: cumulative screening participation in 9 screening rounds (screening round 2 to 10) followed by first screening participation

First screening participation rates did not show significant changes over calendar years (fig 2, top). Compared with participants, first screening non-participants were less likely to participate in future screenings (fig 2, bottom). Over the course of 10 screening invitations, women who participated in the first screening attended an average of 8.74 (95% confidence interval (CI) 8.72 to 8.76) screenings (including the first screen), whereas non-participants attended an average of 4.77 (4.73 to 4.81) screenings. After adjustment for population characteristics that were suspected risk factors for screening non-participation, the odds ratio of first screening non-participation on the second screening non-participation was 7.17 (95% CI 7.05 to 7.28), and on the 10th screening participation it was 3.28 (3.16 to 3.40).

### Breast cancer risk and tumour characteristics

**Fig 3:**
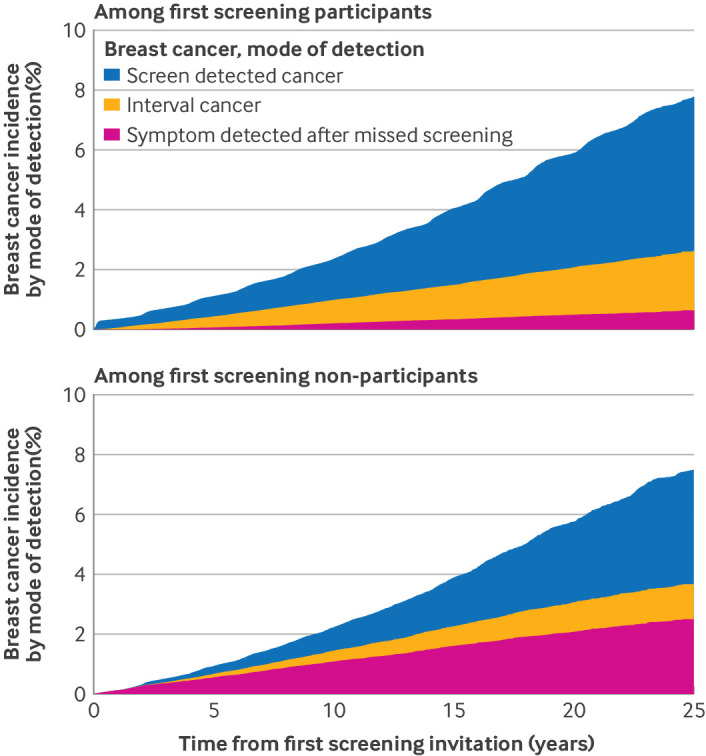
Incidence of mode of detection specific breast cancer, stratified by participation at first invited mammography screening. Stacked cumulative incidences of mode of detection specific breast cancers assessed using Aalan-Johansen estimates.

During a total of 4 940 375 person years of follow-up, we documented 16 059 new cases of breast cancer. The 25 year cumulative incidences of breast cancer did not differ significantly between first screening participants and non-participants (7.8% among participants versus 7.6% among non-participants) (fig 3). However, modes of detection differed markedly: first screening non-participants were considerably more likely to present with symptom detected cancers after missed screening (2.6% *v* 0.7%).

Among women who developed breast cancer, non-participation in the first screening was associated with higher odds of invasiveness (odds ratio 1.32, 95% CI 1.18 to 1.48) and more advanced stage in the multivariable adjusted model. Compared with stage I, the adjusted odds ratio for stage II was 1.19 (1.10 to 1.29), and for stage III and stage IV the odds ratios were 1.53 (1.24 to 1.88) and 3.61 (2.79 to 4.68), respectively (table 2). Cumulative logistic regression also indicated that non-participants tended to have larger tumour sizes (odds ratio 1.32, 1.23 to 1.43) and higher TNM stages (1.34, 1.25 to 1.45).).

**Table 2:** Tumour characteristics of 16 059 women with breast cancer diagnosed during 1991-2020, stratified by participation at first invited mammography screening. Values are numbers (percentages) unless stated otherwise

Tumour characteristics	First mammography screen			Odds ratio (95% confidence interval)	
	Participants (n=11 366)	Non-participants (n=4693)		Partially adjusted model*	Fully adjusted model†
Invasiveness‡:					
In situ tumour	1394 (12.9)	447 (9.9)		1.00 (reference)	1.00 (reference)
Invasive tumour	9420 (87.1)	4047 (90.1)		1.33 (1.19 to 1.49)	1.32 (1.18 to 1.48)
Tumour size:					
<20 mm	6181 (65.7)	2403 (59.4)		1.00 (reference)	1.00 (reference)
20-50 mm	2741 (29.1)	1286 (31.8)		1.21 (1.11 to 1.31)	1.19 (1.10 to 1.29)
>50 mm	486 (5.2)	354 (8.8)		1.88 (1.63 to 2.17)	1.77 (1.54 to 2.03)
Lymph node involvement:					
Negative	6246 (69.6)	2443 (65.6)		1.00 (reference)	1.00 (reference)
Positive	2722 (30.4)	1279 (34.4)		1.20 (1.10 to 1.30)	1.18 (1.09 to 1.28)
Metastases:					
No	9264 (98.9)	3854 (96.3)		1.00 (reference)	1.00 (reference)
Yes	105 (1.1)	150 (3.7)		3.44 (2.68 to 4.43)	3.20 (2.48 to 4.14)
TNM stage:					
I	4634 (51.1)	1735 (44.8)		1.00 (reference)	1.00 (reference)
II	4063 (44.8)	1824 (47.1)		1.20 (1.11 to 1.30)	1.19 (1.10 to 1.29)
III	266 (2.9)	160 (4.1)		1.61 (1.31 to 1.97)	1.53 (1.24 to 1.88)
IV	105 (1.2)	150 (3.9)		3.82 (2.96 to 4.93)	3.61 (2.79 to 4.68)

Odds ratios and 95% confidence intervals are estimated from logistic or multinomial logistic regression models with tumour characteristics as outcome variables.

Total numbers vary across characteristics owing to missing data (missingness ranged from 0.1% to 5.8%).

* Model adjusted for age and calendar year of breast cancer diagnosis.

† Model further adjusted for other covariates including educational attainment, income, migration status, marital status, number of children, age at first birth, alcohol related disease, obesity related disease, Charlson Comorbidity Index, family history of cancer, and family history of breast cancer.

‡ Patients with breast cancer in situ are included only in analysis of invasiveness.

### Mortality

**Fig 4:**
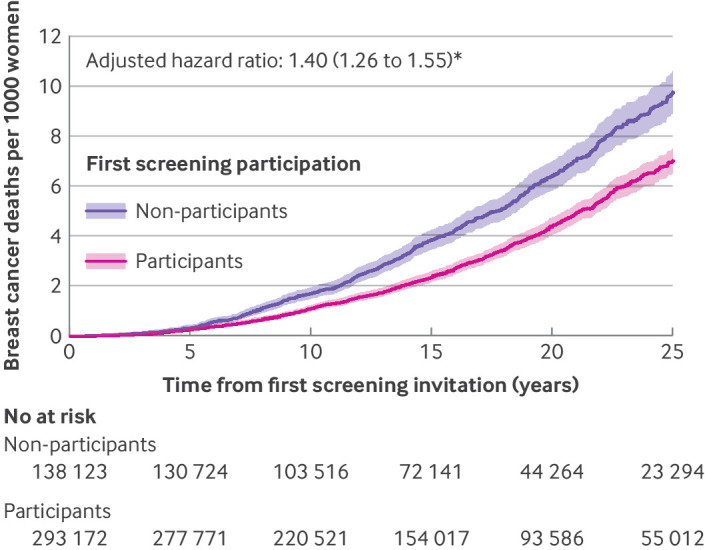
Breast cancer mortality up to 25 years after first screening invitation during 1991-2023, stratified by participation at first invited mammography screening. *Estimates derived from Cox proportional hazard model, adjusted for age and calendar year of first screening participation, educational attainment, income, migration status, marital status, number of children, age of first birth, alcohol related disease, obesity related disease, Charlson Comorbidity Index, family history of cancer, and family history of breast cancer

During a total of 6 818 686 person years of follow-up, we documented 1603 deaths from breast cancer. The 25 year cumulative incidence of breast cancer mortality was significantly higher among first screening non-participants than among participants (9.9 *v* 7.0 per 1000 women) (fig 4). This difference remained robust after adjustment for potential confounders. The hazard ratio for breast cancer mortality among non-participants was 1.46 (95% CI 1.32 to 1.61) in the partially adjusted model and 1.40 (1.26 to 1.55) in the fully adjusted model. Notably, this fully adjusted hazard ratio for breast cancer mortality was higher than the corresponding hazard ratios observed for non-breast cancer mortality (1.27, 1.24 to 1.31) and all cause mortality (1.28, 1.25 to 1.31).

### Sensitivity analysis

We found similar results for breast cancer mortality as in the main analysis when we did sensitivity analyses using age as time scale (hazard ratio 1.41, 95% CI 1.28 to 1.56), excluding women with strong family history (1.42, 1.28 to 1.57), excluding breast cancer cases diagnosed within three months after the screening was missed (1.43, 1.29 to 1.58), and excluding screen detected breast cancers with clinical findings at recall (1.43, 1.30 to 1.59).

## Discussion

Women who did not attend their first mammography screening had a higher mortality from breast cancer than did those who attended; this is probably explained by a lower participation rate in subsequent screenings, which in turn leads to a higher proportion of symptom detected breast cancers (after missed screening), less favourable tumour characteristics, and a worse prognosis. These results emphasise the critical role of first screening attendance in predicting long term outcomes and provide new insights into how early non-participation may be linked to a trajectory of factors that adversely influence breast cancer prognosis.

### Possible explanations for study findings

We found increased breast cancer mortality among first screening non-participants, and we hypothesise that this is primarily due to later detection. Supporting our hypothesis, firstly, we found no evidence of a higher overall breast cancer incidence among non-participants. Secondly, non-participants were significantly more likely to have symptom detected breast cancers diagnosed after a missed screening, which are typically detected at later stages and linked to poorer prognoses.^9^ Additionally, they were more likely to present with advanced stage tumours at diagnosis, which are associated with significantly lower survival rates.^26^ These findings align with previous research showing that delayed diagnosis is a major contributor to breast cancer mortality.^27^ ^28^

A potential alternative explanation for our findings is healthy user (screenee) bias,^29^ ^30^ whereby non-participants may differ from participants in baseline health status or health seeking behaviours, potentially inflating observed mortality differences.  Although the difference in breast cancer mortality that we observed matches or even exceeds that reported in Swedish randomised clinical trials,^7^ ^8^ ^31^ suggesting the possibility of such bias, our analyses suggest that this bias, although present, does not fully explain the observed mortality difference. Notably, the hazard ratio for breast cancer mortality among non-participants (1.40, 95 % CI 1.26 to 1.55) was markedly larger than the corresponding estimate for non-breast cancer mortality (1.27, 1.24 to 1.31), pointing to a disease specific effect rather than a general survival disadvantage.^30^ Furthermore, the association between first screening non-participation and more advanced tumour stages at diagnosis remained largely unchanged after adjustment for factors related to healthy user bias in our fully adjusted model, reinforcing the role of delayed detection. Collectively, these findings provide compelling evidence that delayed diagnosis contributes to breast cancer mortality being higher among first screening non-participants and that the association is not solely due to healthy user bias.

### Comparison with traditional risk factors

Although we observed that the distribution of certain characteristics, such as levels of income, education, marital status, parity, and age at first birth, differed between participants and non-participants, these characteristics are often non-modifiable and unlikely to be altered solely to improve participation in screening. By contrast, first screening non-participation is a modifiable behavioural factor with strong prognostic significance. Even after adjustment for sociodemographic influences, non-participants had a greater than sevenfold higher risk of missing the second screen and a threefold higher risk of missing the 10th, making it the most powerful predictor of long term non-participation in organised screening systems. In addition, unlike many traditional risk factors that need specialised assessments, biomarker testing, or additional questionnaires to identify people at high risk, information on first screening participation is already routinely captured in existing healthcare systems. This practical advantage means that identifying this high risk group requires no additional resources or infrastructure—the data are immediately available at the point of first invitation and response.

### Clinical implications of study findings

The practice changing message of our study is clear: non-participation at the first screening should be prioritised as an early, actionable predictor of avoidable breast cancer mortality. Screening programmes remain insufficiently responsive to this high risk group, allowing persistent disengagement from preventive care and increasing the likelihood of late stage diagnoses and death. This represents a critical missed opportunity for intervention.

According to the 25 year follow-up data, first screening non-participation is not just a transient behaviour but a sign of persistent disengagement from preventive care. This extended time window between identification and the mortality outcome provides healthcare systems with ample opportunity to implement interventions that could disrupt the cascade of poorer screening attendance, delayed detection, and ultimately higher mortality that we observed in this group.

Critically, our results underscore that passive invitation policies, which do not include reminders or follow-up for non-attendees, leave first screening non-participants largely untouched and therefore disproportionately exposed to excess breast cancer mortality. Randomised trials have shown that simple, low cost interventions—such as automatically scheduling a second, fixed date appointment or offering brief telephone coaching^32^ ^33^—can markedly improve mammography screening among non-participants. By showing that first screening non-participants represent a persistently disengaged group with a 40% higher risk of breast cancer mortality, our study provides the essential justification for integrating these proactive strategies into routine mammography screening practice.

### Strengths and limitations of study

A key strength of this study lies in the use of population based data from the Swedish Mammography Screening Register and linked Swedish nationwide registers. The unique personal identity number system in Sweden enabled us to follow up, in theory, every participant in our study population for up to 25 years. This comprehensive follow-up benefitted from robust register systems, minimised the risk of loss to follow-up, and ensured the accuracy and completeness of our data. Additionally, detailed information on screening participation, tumour characteristics, and mortality allowed for a thorough analysis of long term breast cancer outcomes.

However, despite adjustment for sociodemographic, reproductive, and health related factors, residual confounding from unmeasured psychosocial and behavioural factors cannot be ruled out, precluding causal inference about the effect of mammography screening on breast cancer mortality. Secondly, the Swedish Mammography Screening Register does not capture breast cancer detection or imaging done outside the organised programme, such as in private clinics or through alternative methods such as ultrasonography or magnetic resonance imaging. These instances are rare in Sweden and typically limited to women at high risk, such as those referred for genetic counselling because of a strong family history of breast cancer. We accounted for this potential source of bias by doing sensitivity analyses excluding women with a family history of breast cancer; findings remained consistent. Finally, the cohort comprised women in Sweden, a country with structured, longstanding screening protocols. Findings may not fully generalise to populations with different healthcare systems, screening intervals, or cultural attitudes towards preventive cares.

### Conclusions

Our study shows that first screening non-participants represent a large population at an elevated risk of dying from breast cancer decades in advance. This increased mortality is modifiable and primarily attributed to late detection. Targeted interventions are warranted to boost adherence to mammography screening and decrease the mortality risk for those who did not participate in the first screening. In addition, our findings shed light on implications not only for breast cancer but also for other cancer screening programmes.

## What is already known on this topic

Evidence suggests that mammography screening reduces breast cancer mortality, although debate persists
Breast cancers detected among non-participants in mammography screening have worse tumour characteristics than those in participants

## What this study adds

First screening non-participants had a 40% higher breast cancer mortality risk than participants, persisting over 25 years
The increased mortality is mainly due to delayed detection of breast cancer
Targeting first screening non-participants (32.1% of invited women) offers a critical opportunity to reduce breast cancer mortality at the population level

## Data Availability

Owing to privacy protection, including the EU’s General Data Protection Regulation (GDRP), the (de-identified) individual level data from Swedish healthcare registers used in this study cannot be publicly shared. Access requires ethical approval and compliance with Swedish legislation; interested researchers must request access directly from the relevant Swedish healthcare data holders. The study protocols and statistical analysis plan are available on reasonable request to the corresponding author. The analysis code is openly accessible at https://github.com/ziyan-ma/FirstMammogram.

## Notes

Contributors: ZM, WH, and KC conceived and designed the study. ZM, WH, YZ, JT, KH, and KC acquired, analysed, or interpreted the data. PH provided clinical expertise. ZM and YZ did statistical analyses. ZM, XM, and JT were responsible for data management. KC provided administrative, technical, or material support. ZM, WH, and KC drafted the manuscript. All authors contributed to the revision of the manuscript and approved the final draft. ChatGPT was used to improve fluency and provide proofreading assistance. KC is the guarantor. The corresponding author attests that all listed authors meet authorship criteria and that no others meeting the criteria have been omitted.

Transparency: The lead author (the manuscript’s guarantor) affirms that the manuscript is an honest, accurate, and transparent account of the study being reported; that no important aspects of the study have been omitted; and that any discrepancies from the study as planned (and, if relevant, registered) have been explained.

Dissemination to participants and related patient and public communities: The findings from this study will be disseminated through multiple channels to reach both healthcare professionals and the general public. A plain language summary in Swedish is being prepared for publication on the KARMA (Karolinska Mammography Project for Risk Prediction of Breast Cancer) website (www.karmastudien.se) to ensure accessibility for participants and interested members of the public. Following academic publication, we will collaborate with the Karolinska Institutet press office to issue a press release aimed at media outlets and public health communication platforms to enhance public engagement and awareness. Additionally, the study results will be shared with collaborators within the Breast Cancer Association Consortium (BCAC), an international network of more than 100 research groups dedicated to investigating risk factors for breast cancer. Dissemination within this forum will support continued scientific dialogue and collaborative progress.

Provenance and peer review: Not commissioned; externally peer reviewed.
