# EXPRESSION OF CONCERN: First mammography screening participation and breast cancer incidence and mortality in the subsequent 25 years: population based cohort study

**DOI:** 10.1136/bmj.r2394

**Published:** 2025-11-25

## What happened after publication

After publication of this paper by Ma and colleagues (*BMJ* 2025;390:e085029, doi:10.1136/bmj-2025-085029, published 24 September 2025) and its linked editorial (*BMJ* 2025;390:r1893, doi:10.1136/bmj.r1893),^1^ ^2^ BMJ was alerted to concerns that messaging in key areas may not be sufficiently supported by the data presented in the work.

## Reason for expressing concern

The research paper^1^ shows an association between non-attendance at first breast screening and death from breast cancer. There is concern that a lack of data about all cause mortality, and/or lack of emphasis on those data, is a critical limitation. This may impact the implications of the work and BMJ is conducting additional statistical review.

The main finding of the paper is an association between attendance at first breast screening appointment and breast cancer death. Both the authors of the research paper and the editorial conclude and/or call for interventions to improve adherence to screening. Their call not only implies a causal link but one which is modifiable using interventions to increase screening. The call is not sufficiently grounded in the conclusions of the data analysed in this paper.

## Further action

BMJ is in discussion with the authors about what post-publication change to their work is required to ensure that it accurately reflects the results and other relevant evidence, and is transparent about uncertainties.
